# Improved strategy for the curation and classification of kinases, with broad applicability to other eukaryotic protein groups

**DOI:** 10.1038/s41598-018-25020-8

**Published:** 2018-05-01

**Authors:** Andreas J. Stroehlein, Neil D. Young, Robin B. Gasser

**Affiliations:** Melbourne Veterinary School, Department of Veterinary Biosciences, Faculty of Veterinary and Agricultural Sciences, The University of Melbourne, Parkville, Victoria, 3010 Australia

## Abstract

Despite the substantial amount of genomic and transcriptomic data available for a wide range of eukaryotic organisms, most genomes are still in a draft state and can have inaccurate gene predictions. To gain a sound understanding of the biology of an organism, it is crucial that inferred protein sequences are accurately identified and annotated. However, this can be challenging to achieve, particularly for organisms such as parasitic worms (helminths), as most gene prediction approaches do not account for substantial phylogenetic divergence from model organisms, such as *Caenorhabditis elegans* and *Drosophila melanogaster*, whose genomes are well-curated. In this paper, we describe a bioinformatic strategy for the curation of gene families and subsequent annotation of encoded proteins. This strategy relies on pairwise gene curation between at least two closely related species using genomic and transcriptomic data sets, and is built on recent work on kinase complements of parasitic worms. Here, we discuss salient technical aspects of this strategy and its implications for the curation of protein families more generally.

## Introduction

In the last decade, the number of available nucleotide data sets has increased substantially, enabled by major advances in sequencing technologies^1–5^. For these data to be useful biologically, they need to be represented by high-quality genomes and transcriptomes, which need to be annotated. Accurate annotation requires a reliable and, preferably, automated procedure for the identification of genes and classification of inferred protein sequences. Although many software packages and tools have been developed for this purpose^6^, accurate annotation remains a challenge, particularly for most eukaryotes^7^. Nevertheless, algorithms allow automatic searches and comparisons of biological sequences, as well as the identification of conserved patterns and/or domains^8^. Such algorithms have substantially increased the ability to infer the relatedness of sequences and predict their biological functions^9,10^.

Historically, the prediction of protein function and/or the classification of proteins have relied on sequence similarity searches against protein sequence databases using the Basic Local Alignment Search Tool (BLAST)^11,12^. In this context, sequences are often compared with those of model organisms (such as *Caenorhabditis elegans* and *Drosophila melanogaster*), for which experimental evidence of protein function is available. However, for organisms that are genetically very distinct from model organisms, such comparisons often result in limited sequence similarity and inaccurate annotations^13,14^.

To address this limitation, stochastic models, such as hidden Markov models (HMMs) and position-specific scoring matrices (PSSMs), have been developed^15–17^. These models allow sensitive and specific searches for conserved functional domains and have been implemented in many tools, including those employed to identify protein kinases^18,19^. HMMs are statistical descriptors of sequence similarity inferred from multiple sequence alignments (MSAs), and have been shown to be more sensitive and specific than local alignment-based methods for sequence searches and/or comparisons, such as BLAST^20^. However, most HMMs have been constructed using ‘seed’ MSAs that poorly represent non-model organisms and, thus, might not be sensitive enough to detect divergent but related sequences.

**Figure 1:**
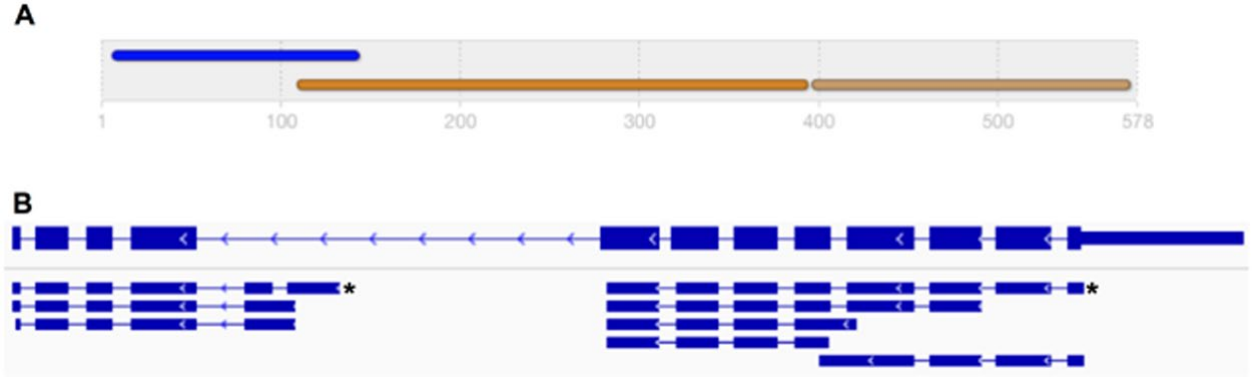
A protein sequence that was incorrectly inferred from an erroneous gene prediction (M514_09783) in the draft gene set of the *Trichuris suis* genome (adult female; PRJNA208416). (**A**) Amino acid domain architecture (based on InterProScan^46^) of the inferred “protein kinase/CAP” fusion protein, containing domains typical of protein kinases (“SH2 domain”; blue; IPR000980 and “protein kinase domain”; orange-brown; IPR000719) and a CAP domain (light brown; IPR014044). (**B**) Original gene model for M514_09783 on *scaffold71* (accession number: KL367486) of the genome assembly of *T. suis* (top); exons are displayed as thick blue boxes, introns as blue lines and the 5′ untranslated region as a thin blue box. Eight transcripts (*de novo*-assembled from publicly available RNA-Seq data) are mapped to *scaffold71* in this region (below), refuting the original gene prediction and providing evidence for two independent gene models. The longest transcript isoforms represent the curated gene models and are indicated by asterisks.

In addition to challenges in identifying and annotating protein sequences, some gene prediction approaches can inaccurately infer gene models due to, for example, incomplete or fragmented genome assemblies^21^ or genes being located close to each other in the genome^22,23^. Incorrect predictions can have a substantial impact on subsequent analyses. For example, the identification of a protein that is represented by an incorrect combination of two adjacent gene models (‘fusion’; Fig. 1) might fail, because the stochastic model (i.e. HMM or PSSM) built to detect a characteristic protein domain yields a low-confidence score. Similarly, for a sequence correctly identified based on a conserved domain, incorrectly predicted N- or C-terminal regions might lead to a misclassification, or preclude a classification based on sequence similarity. Importantly, inaccurate nucleotide and/or protein sequence data can lead to errors in the annotation of biological pathways^24^ and in analyses of differential expression^25^.

To assess the accuracies of gene predictions and functional annotation of protein sequences achieved by ‘best-practice’ bioinformatic pipelines^6^, we recently investigated the protein kinase complements (kinomes) of a range of parasitic worms (helminths, including flatworms and roundworms)^26–29^ for which genomes and transcriptomes are available. Despite the expansion of such data sets for parasitic worms^30^ and the importance of protein kinases in a plethora of key biochemical signalling pathways^31–33^, very little is known about protein kinases encoded in these genomes and their functions. Fortunately, protein kinases contain a well-conserved catalytic domain whose sequence and biochemical mechanisms have been studied extensively in most model organisms, such as *Saccharomyces cerevisiae*^34^, *Homo sapiens*^35^, *C. elegans* and *D. melanogaster*^31^. The wealth of curated literature and data on kinases can be harnessed to curate respective genes, and to characterise kinomes of parasites.

Elucidating kinomes of parasites will provide new insights into the biology of these unique organisms. In this context, the biological and phylogenetic differences between parasitic eukaryotic organisms and well-studied free-living model eukaryotes can provide clues regarding the evolution of parasitism. In addition, protein kinases have also been extensively investigated as drug targets for human diseases, such as cancers, and are amenable to targeting by small molecule drugs^36–38^, providing promising avenues for the control of parasitic worms^39–41^. Taken together, there is a need to establish an approach for the accurate annotation of protein kinases of parasitic worms.

Several tools and resources for the identification and classification of protein kinases are available, including the programs Kinomer^18^ and Kinannote^19^. These tools rely on a set of kinase-specific HMMs, PSSMs and/or BLAST searches against curated kinase databases. For instance, Kinannote takes approximately five minutes to run on a protein data set of 10,000–15,000 sequences and is fully automated. Furthermore, it produces a draft kinome and comparative statistics of the analysed kinome, and is the only tool that automatically classifies protein kinases (including some atypical kinases) using the currently recognised classification scheme for these enzymes^42^.

However, there are challenges associated with the use of such tools. Firstly, automated protein identification and classification rely on the use of evidence from either protein domain annotation or sequence comparison; decisions based on such evidence are sometimes made in a ‘black box’, without the user being able to assess intermediate results or adapt the workflow to individual requirements (e.g., cut-off scores or the use of specialised models/databases). Secondly, the models employed to identify and classify sequences often do not include or consider divergent sequences (e.g., those of helminth species), thus hampering reliable detection. Additionally, most protein identification/classification tools accept protein sequence data as input, but do not permit the use of RNA-sequencing (RNA-Seq) evidence or genomic data, which could aid in the curation process. Inaccurate *in silico* gene predictions from draft genomes will preclude the accurate identification and classification of protein kinase sequences.

Thus, none of presently available tools achieves a comprehensive curation of a kinome. Furthermore, current tools do not adequately consider the fragmentation of draft genomes and the substantial kinome diversity between, for instance, the well-curated model organism *C. elegans*^43^ and other, distantly related worms - this represents a challenge, both for the identification and the classification of protein kinase sequences in these genomes. Moreover, none of the currently available tools provides outputs that allow for the detailed, semi- or fully automated curation of nucleotide/amino acid sequences inferred from draft genomes. These limitations show that these tools are not universally applicable to kinomes of eukaryotes.

Here, we describe a strategy for the curation of gene models and subsequent annotation of inferred protein sequences for protein families of non-model organisms, based on recent studies of kinase genes of parasitic organisms. This strategy is continually being improved to tackle some of the inherent challenges in analysing different genomic and transcriptomic data sets.

## Curation and annotation strategy

### Defining and curating gene families

**Figure 2:**
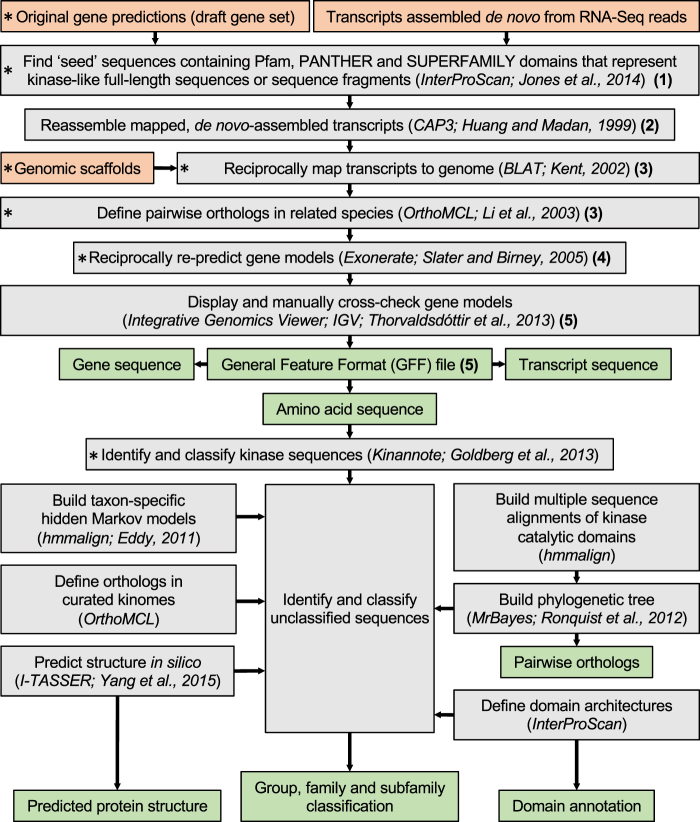
A strategy for pairwise gene curation, kinase identification, classification and functional annotation. Orange boxes represent input data, green boxes represent output data and grey boxes specify individual steps, with employed programs and references given in brackets for each step. Essential steps in this workflow are marked with an asterisk (left); the five individual gene curation steps (1 to 5). Following this workflow, the output data can be employed as input for subsequent analyses such as pathway annotation, drug target prediction/prioritisation and/or the analysis of transcription profiles of kinase genes.

The gene curation strategy integrates draft genomic and/or transcriptomic data from two or more species, and is divided into five distinct steps (Fig. 2):

#### Identification of candidate sequences based on characteristic functional domains

First, all amino acid sequences (encoded within a draft genome) that contain one or more conserved functional domains characteristic of a particular protein (super-)family (e.g., the catalytic domain representing protein kinases), are identified employing information from domain matches against the databases Pfam v.27.0 (ref.^20^), PANTHER v.9.0 (ref.^44^) and SUPERFAMILY v.1.75 (ref.^45^) within the program InterProScan v.5.15.54 (ref.^46^). As this first step identifies candidate sequences, stringent cut-off values (e.g., E-value of ≤10^−5^) are not applied to not miss related but divergent sequences. Importantly, accessory domains that are commonly found in this protein family are also considered as candidate sequences. For example, in the case of protein kinases, sequences that lack a conserved kinase catalytic domain but contain one or more SH2 domains (accessory domain/s commonly present in tyrosine kinases) might represent fragments of a protein kinase sequence. Such fragments can be highly informative and essential for curating a final gene model. Accordingly, every sequence identified that contains at least one characteristic conserved domain represents a ‘seed’ that can be extended N- and C-terminally in subsequent steps, thus complementing the underlying draft gene model. Additionally, to provide a reference data set, sequences in databases representing the investigated protein family are interrogated in the same way. For studies of kinases, we use all domain combinations present in curated protein kinase complements (i.e. those deposited in the KinBase database; http://www.kinase.com/kinbase/) and new domain architectures as they become available. We also include subsets of the identified domains as candidates, reasoning that they might represent sequence fragments that can be joined into a complete gene model during the curation process.

#### Mapping and reassembly of ‘seed’ transcripts

If *de novo*-assembled transcripts (i.e. assembled using RNA-Seq reads, independent of a reference genome) are available, all transcripts are mapped to genomic scaffolds using the program BLAT v.34 × 12 (ref.^47^). Then, all mapped transcripts overlapping with a region to which at least one candidate ‘seed’ transcript has been mapped are reassembled using CAP3 (build 15 Oct 2007)^48^. The longest, reassembled transcript sequence containing an open reading frame (ORF) is then selected for the subsequent re-prediction of its gene model.

#### Mapping transcripts to related genome sequences and defining pairwise orthologs

Both transcripts identified from existing gene sets and extended, reassembled transcripts are mapped to a genome sequence of a phylogenetically closely related species. In this context, it is important to carefully select the related species, and ensure that high-quality genomic and transcriptomic data are available. On one hand, the selection of two, phylogenetically divergent species might lead to a lack of comparative genomic and transcriptomic evidence needed for curation. On the other hand, two closely related species might not add any complementary information to the re-prediction process. Based on this mapping step, the most closely related ortholog in the related species can be identified, forming pairs of sequences for subsequent curation. The pairwise orthology is then confirmed using tools such as OrthoMCL v.2.0.4 (ref.^49^) or an all-against-all pairwise global alignment (e.g., EMBOSS Needle v.6.3.1)^50^.

#### Reciprocal re-prediction of gene models

The “coding2genome” model in the program Exonerate v.2.2.0 (ref.^51^) is used to define gene structures (i.e. intron-exon boundaries) based on mapped transcripts encoding candidate proteins. This step is then reciprocally iterated between the two genomes, being compared until an optimal gene prediction is achieved for pairs of orthologs in both genomes (i.e. the prediction converges).

#### Quality assessment and output

**Figure 3:**
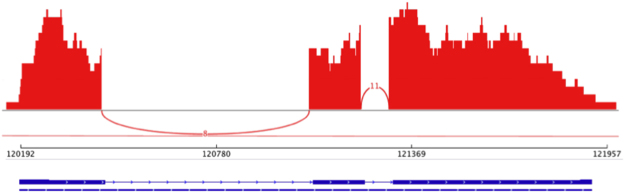
Example of a Sashimi plot (red). Depicted are the coverage of mapped RNA-Seq reads and the number of paired-end reads supporting intron-exon boundaries for a gene model (blue).

Curated gene models are assessed for an ORF using the program getorf (within the EMBOSS package v.6.4.0.0)^50^. Additionally, mapped transcriptomic reads can be used to confirm that intron-exon boundaries have been correctly identified for the curated gene models (e.g., using Sashimi plots; Fig. 3). Next, candidates with insufficient or inconclusive domain evidence are discarded. A length cut-off can be applied, if a particular gene/protein family is known to have a minimum length. For example, for protein kinases we choose a minimum length cut-off of 200 amino acids. Sequences that do not fulfil these criteria can either be excluded from further analyses or labelled as fragments. Then, the final curated gene sets and their genomic loci are reported in a “general feature format” (GFF) file for each investigated genome data set.

### Classification and functional annotation

For protein kinase classification, we employ a sequence similarity (BLAST) search against KinBase within Kinannote. Additionally, for sequences not (fully) classified by Kinannote, we use OrthoMCL (BLAST E-value of ≤10^−5^; sequence similarity of ≥80%) to cluster sequences from the species being studied, *C. elegans* and *H. sapiens*, and subsequently infer classification based on the annotation of the curated sequences in the latter two species^27,28^. Sequences that cannot be categorised using these two approaches are classified based on the presence and order of functional domains and/or signatures (“domain architecture”) as determined by InterProScan. However, the family/subfamily-specific HMMs within the Pfam, PANTHER and/or SUPERFAMILY databases employed for this task are not always representative of the diversity across the Tree of Life^52^, being built based on a relatively small number of well-characterised sequences. Thus, we employ specialised models built specifically for the classification of particular protein families^26,53,54^, which are more sensitive for selected taxa than related models in established databases.

In addition to sequence-based classification, we employ three-dimensional structural modelling of unclassified sequences using solved crystal structures deposited in the Protein Data Bank (PDB; http://www.rcsb.org/pdb/), and by employing the program I-TASSER v.4.4 (ref.^55^) for *de novo* predictions. Stringent cut-offs are applied (similar to an E-value cut-off for sequence similarity-based annotation approaches) to ensure high confidence structural predictions and associated classifications. For example, for kinase classification, we only consider the structurally most similar entry in the PDB and apply cut-offs for template modelling (TM) scores (≥0.75), confidence (C) scores (>0) and root-mean-square deviation (RMSD) values (<3 Å).

Following classification, functional annotation is achieved by assigning domain identifiers and gene ontology (GO) terms to sequences and by linking them to biochemical pathways based on similarity (employing protein BLAST v.2.2.28+; E-value of ≤10^−5^; ref.^12^) to sequences in the Kyoto Encyclopedia of Genes and Genomes (KEGG) database^56^. The predicted subcellular localisation of proteins is inferred using the program MultiLoc2 (ref.^57^), applying a conservative cut-off of ≥0.8 for the confidence score. Additionally, transcription analysis in particular developmental stages or different tissues can aid functional annotation. However, as the present article focuses on sequence- and structure-based annotation, we refer the reader to two recent publications^27,29^ for more information on transcription analysis as a tool for annotation.

### Confirming classification and pairwise orthology

The inferred classification and pairwise orthology of sequences from different species are confirmed by phylogenetic analysis^28^ employing the program Mr. Bayes v.3.2.2 (ref.^58^). In this context, it is important to carefully select the part of the sequences that should be used for the construction of MSAs underlying the phylogenetic trees. For example, to achieve robust trees (i.e. with high nodal support values), in kinase studies, we only use the catalytic domains of protein kinases and analyse all nine recognised kinase groups independently.

## Discussion of experiences made while establishing the curation strategy

### Improved and automated gene family curation

The applicability, robustness and usefulness of the established curation strategy described here is exemplified by several recent studies that have curated the gene models encoding protein kinases in the genomes of some key parasitic worms (including species of *Haemonchus*, *Trichinella*, *Trichuris* and *Schistosoma*), and classified and functionally annotated the kinomes of these worms^26–29^.

Some published tools/strategies follow similar approaches. For example, a recent study^25^ also employed a reciprocal re-annotation approach of genes using Exonerate to improve gene models for subsequent differential expression analysis. The tool OrthoFiller^59^ employs HMMs, built from clusters of orthologous proteins, to improve gene predictions in draft genomes. Additionally, the program GeneValidator^60^ automatically identifies erroneous gene models in existing draft genomes and tags them for subsequent manual curation. Similarly, the database MisPred^61^ utilises deviations in domain size and architecture and a range of other sequence characteristics to detect incorrect gene models and infers proteins in public databases for 19 metazoan ‘model’ organisms. Subsequently, the FixPred pipeline^62^ attempts to correct sequences identified by MisPred *via* database searches, reassembly of fragments/RNA sequences or *de novo* re-prediction of a gene model. Other approaches improve *de novo*^63^ and reference-guided^64^ transcriptome assemblies by combining the outputs of multiple different assembly approaches, thus achieving improved assembly metrics and reducing the number of fragmented and/or incorrectly fused transcripts sequences.

We have introduced several additional steps to achieve improved gene prediction. For example, the use of extensive transcriptomic data sets allowed for the application of an improved gene re-prediction approach. Additionally, the automated re-assembly of *de novo*-assembled transcripts mapped to the genomic scaffolds (using CAP3) and subsequent inference of ORFs accelerated the detection and correction of erroneous gene predictions, and the confirmation of correct ones. However, this approach relied on the availability of extensive RNA-Seq data. In cases where such data were not available or were of insufficient quality/quantity, the re-prediction of gene models was achieved by transferring re-assembled, high-confidence gene models from one species to a closely related one^27^.

In addition to re-predicting gene models based on re-assembly of transcriptomic data, the location of genes on genomic scaffolds allowed the inference of a range of other key features that were used in the curation and/or annotation process. For example, information about intergenic distances and the completeness of protein domains encoded by adjacent genes was employed to complement fragmented genes and curate incorrect splice sites that were introduced during gene prediction and/or scaffold assembly. Furthermore, analysing predicted functional domains and comparing them with known architectures of functional domains in multiple organisms helped to identify and curate fragmented and/or incorrectly assembled genes.

Employing this approach for the curation of worm kinomes, the considered selection of two species for pairwise curation and the use of additional, independent data sets (e.g., those created employing distinct methodologies) allowed ‘consensus’ gene models to be created from all data sets. Using this strategy, we have overcome some of the challenges inherent to draft genome assemblies and inferred high-confidence gene models.

### Manual curation

**Figure 4:**
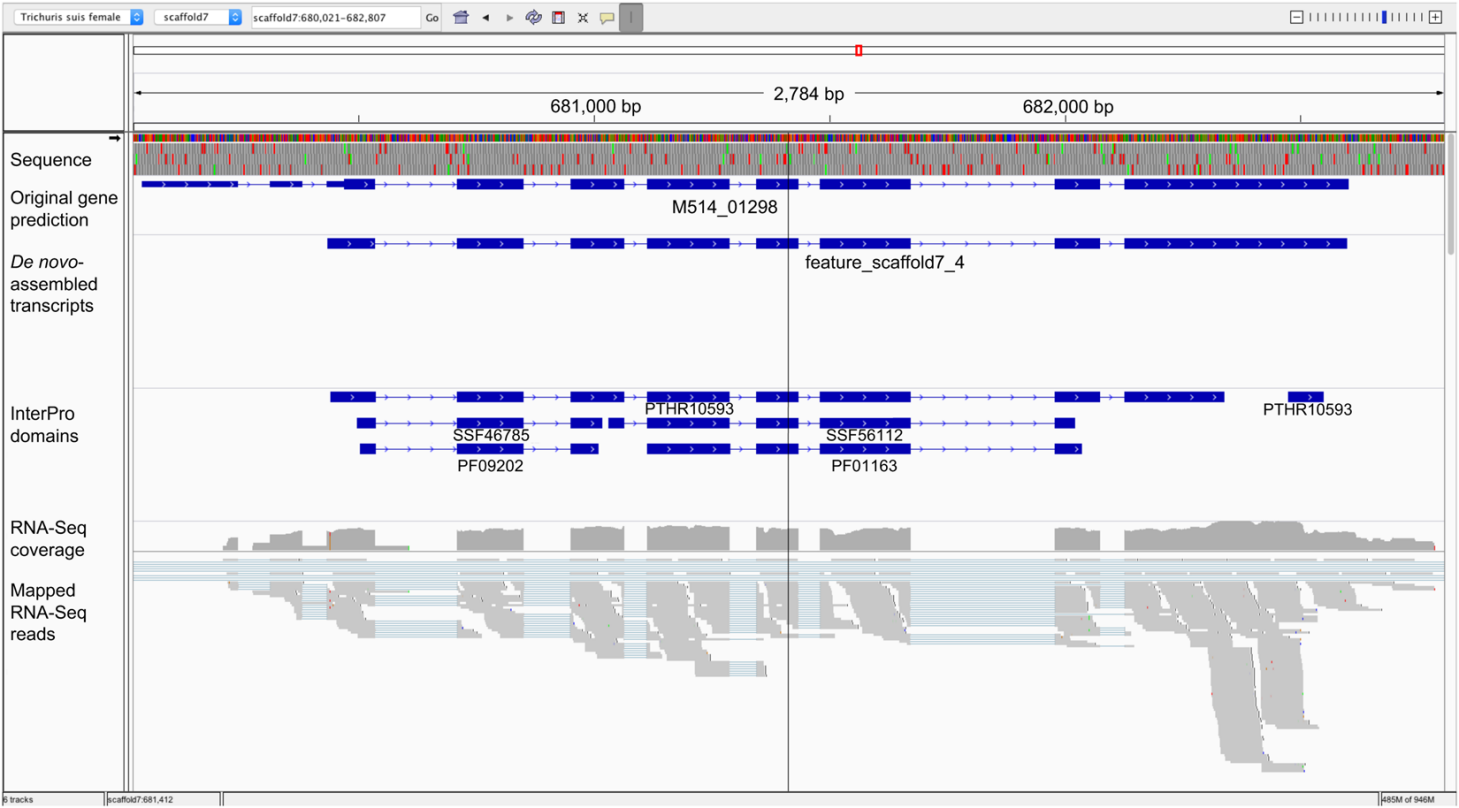
A typical window in the Integrative Genomics Viewer (IGV) software^65^. Displayed are a 2784 base pair (bp) section of *scaffold7* of the *Trichuris suis* genome (adult female; PRJNA208416), an original gene prediction (M514_01298), a mapped *de novo*-assembled transcript representing the curated gene model (feature_scaffold7_4), mapped functional domain annotations (PTHR10593, SSF46785, SSF56112, PF09202, PF01163) inferred using InterProScan^46^, and mapped paired-end RNA-Seq reads.

In addition to advances in the automated re-prediction of gene models, manual and/or semi-automated curation of the associated genomic and transcriptomic data was carried out to confirm correct gene predictions. To this end, a genomic viewer such as the Integrative Genomics Viewer (IGV)^65^ was an indispensable tool for the simultaneous display of gene models, genomic locations and complementary data, including functional domains, RNA-Seq support and *de novo*-assembled transcripts (Fig. 4).

In our experience, manual curation helped identify ‘hotspots’ of potentially incorrect predictions, such as mis-assembled genes. For example, the gene prediction software originally employed in the genome projects (MAKER2; ref.^66^) tended to splice two or more genes with short intergenic distances to each other, to produce a single gene model, when, in fact, they represented separate genes (Fig. 1). This phenomenon might have been caused by the compactness of worm genomes, which contain small intergenic regions and overlapping genes^67–69^. In our experience, the use of IGV, *de novo*-assembled transcripts and mapped paired-end RNA-Seq reads could refute or support such gene predictions (Figs 1,3 and 4)^27,28^; gene models were suggestive of being incorrect if no *de novo*-assembled transcript spanned the entire length of the predicted gene and if mapped paired-end reads did not support (i.e. span) intronic regions. In contrast, most other gene models were automatically confirmed either using transcriptomic evidence for the same species or, employing our automated curation strategy, based on transcripts assembled for closely related species.

### Curated gene models enable enhanced classification and functional annotation

Gene curation represents a critical component of any annotation workflow, because all subsequent identification, classification and annotation steps rely on the quality of the underlying gene prediction. To achieve reliable protein annotation at the primary sequence and structural levels, a gene needs to have transcriptomic support, an accurate genomic location and an ORF. The curation strategy presented here provides the basis for high-confidence protein annotation, which, in our studies, was achieved using Kinannote and an orthology-/phylogeny-based approach^26–28^.

The pairwise comparison approach applied for gene-level (i.e. protein-coding sequence) curation allowed an iterative refinement of protein classification and functional annotation through cross-validation of genomic and transcriptomic data with the inferred protein sequences. For every prediction, a consensus was based on the evidence from RNA-Seq data, *de novo*-assembled transcriptomes, predicted protein domains and their architecture, genomic assemblies and gene predictions from published gene sets. These data were collated for two relatively closely related species and, in some cases, for multiple data sets representing the same species^27^ to add additional confidence to predictions. Importantly, the semi-automated analysis of domain architectures helped identify putative fusions (i.e. co-occurrence in the same protein sequence) of domains that do not occur together in other kinase sequences within the InterPro database^27,28^. Such ‘fusions’ were indicative of an erroneously assembled gene sequence, and required critical assessment at the genomic and transcriptomic sequence level to either support (based on RNA-Seq evidence) or refute the predicted model. This proved useful for the correction and complementation of erroneous gene predictions. Taken together, the integration of the different data types resulted in consensus gene predictions that enabled the inference and classification of protein sequences with high confidence.

For kinase sequences from species that were not accurately represented by the HMMs available in public databases, we built sensitive, taxon-specific kinase HMMs to account for sequence diversity among species^26^. However, such an approach might ‘overfit’ the stochastic model to a particular organism, thus losing general specificity for a wide range of protein kinases from other species. Accordingly, curated kinase sequences of worms should be added to the protein kinase HMMs in Pfam, thus building improved models that are more sensitive to phylogenetic distant kinase sequences, while retaining specificity. In this context, it might also be useful to integrate a recently established BLAST-based taxon sampling approach to identify more distantly related (‘hidden’) orthologs^52^.

In previous studies^27,28^, we identified kinase sequences that were phylogenetically distinct from those in recognised families or subfamilies by grouping the predicted catalytic domains of unclassified kinases into orthologous clusters and subsequently constructing phylogenetic trees. Such analyses elucidated the phylogenetic relationship between recognised families and novel, species-specific families that were not detected by tools such as Kinannote. A comparison between the annotation by Kinannote and that of the orthology/phylogeny-based approach revealed kinase sequences that were specific to a phylogenetic branch or even a single genus/species. This approach provided important information about the evolution of protein kinase families/subfamilies and about the potential function of novel kinases. By using a combination of a controlled vocabulary and an orthology-/phylogeny-based approach, most sequences that have diverged from their non-helminth orthologs were automatically assigned to the recognised groups, families and subfamilies.

In addition, the classification of sequences based on functional domain architectures enabled (sub-)grouping of kinases based on the presence and order of functional domains. To date, this approach has been applied solely to the classification of protein kinases. To extend the use of this approach to any protein family, the entire InterPro database would have to be processed in a similar manner. The online version of InterProScan IDA tool allows searches for single architectures but does not permit batch queries for entire proteomes. Furthermore, a locally installed, stand-alone version of the IDA tool is currently not available or planned (InterProScan staff, personal communication). Such a tool would allow searching for sequences with the same or similar domain architecture in a high-throughput manner, thus facilitating the exploration of gains or losses of functional domains in sequences across different phylogenetic taxa.

Lastly, the integration of three-dimensional modelling data enriched the annotation of protein sequences, beyond what would have been possible using primary sequence- and sequence domain-based approaches. For example, in two recent kinome studies^27,28^, this approach facilitated the reliable annotation and classification of a previously uncharacterised kinase in four species of roundworm (phylum Nematoda) within the class Enoplea.

Taken together, the combination of a pairwise reciprocal curation approach, Kinannote, an orthology-/phylogeny-based classification, a domain-based curation and annotation strategy and three-dimensional modelling underpins our improved strategy for the characterisation of kinases and addresses salient limitations of other tools presently available (e.g., Kinomer^18^ and Kinannote^19^). Importantly, although applied to kinases, our practical and transparent strategy should be broadly applicable to the comprehensive annotation of most other protein families.

### Implications of improved gene curation strategies and updated biological sequence databases

The improvement or correction of gene predictions represents an important component of any genome project, and has a substantial impact on subsequent analyses of genomic and transcriptomic data sets. Without iterative cycles of curation of existing genomic and transcriptomic resources, incomplete or incorrect gene predictions for a genome deposited in public repositories will most likely be perpetuated, as data will be utilised as protein evidence (e.g., in the MAKER2 pipeline^7,66^) for *de novo* gene predictions (i.e., those lacking RNA-Seq support) in genome projects of related species. This issue emphasises the need for improved gene prediction software (e.g., the recently released MAKER3 pipeline; v. 3.00.0-beta; http://www.yandell-lab.org/software/maker.html), for the comprehensive curation of erroneous gene predictions (either by individual curators or through research community efforts^70,71^) and importantly, for the integration of curated data into existing repositories.

To enable the integration of data produced using the present strategy, we have provided coding sequences, amino acid sequences and genome locations for all curated kinome data sets. In addition, we have automatically created output files in GFF format; this format can be read by most genomic viewers/editors, such as IGV^65^ or Web Apollo^72^, and allows gene-level annotations to be interactively viewed and edited, and published draft gene sets to be updated in online repositories. The use of an established, generic output format, such as GFF, represents a major advantage over other tools for protein family identification and classification.

The use of a genomic viewer has also facilitated the iterative improvement of the present strategy. Once a new method or functionality was implemented, its performance needed to be assessed by simulating common ‘use cases’ or ‘edge cases’ (i.e. cases that occur rarely but when they occur, are likely to cause the program/algorithm to fail or not perform satisfactorily). This systematic evaluation was aided by manual confirmation of representative gene/transcript features (‘spot checking’) *via* a genomic viewer, and helped confirm the satisfactory performance of the implementation. Such assessments of newly implemented, automated methods identified aspects that might be improved and/or further automated in the future.

For example, the prediction/correction of gene models based on transcript data employing CAP3 and Exonerate^51^ was established in an earlier investigation^28^ and then automated and further improved in a subsequent study^27^. This improvement included an automated, global analysis of donor and acceptor sites of introns, which was then integrated into the gene prediction process in Exonerate, thus improving the accuracy of intron-exon boundary predictions. Although we employed Exonerate to re-predict gene models initially^26^, we carried out multiple individual runs of single genes that needed to be complemented based on manual inspection. These examples show how the curation and annotation strategy was successively adapted to tackle the technical challenges of multiple, distinct data sets, particularly with respect to the quality and amount of data available. The continuous growth of publicly available data sets and data repositories should enable the integration of additional data in the future, and highlights the need for reliable and reproducible gene prediction/annotation workflows that are able to process and analyse such data sets.

Taken together, manual curation and systematic assessment of gene models informed the automation process. In this context, it is important to carefully consider the benefit gained from automating a particular step *versus* the time spent implementing the automation, and the accuracy and speed of the automated method *versus* that achieved by manual curation. It is also important to consider the size of the data set that is to be curated. For example, protein kinases represent a relatively small and well-defined data set (~200–500 sequences) compared with other assemblages of proteins (e.g., all proteins that play a role in metabolic pathways; likely thousands), which allowed for the application of additional, manual curation efforts. Generally, automated steps should only be included in the next iteration of a tool or pipeline if they can be achieved without a loss of accuracy. Importantly, our focus has been to automate steps that, although being algorithmically less challenging (e.g., file format conversions or output-input transformations), often represent steps in a workflow that are most prone to human error. In contrast, other steps proved harder to automate and were more reliable if implemented in a semi-automated manner, with guidance/intervention by an expert. This approach did not affect the ability to process the input in a computer-readable format and allowed decisions to be made and stored for future analyses, achieving a near-optimal or optimal solution after multiple iterations.

### Future focus and improvements of the established strategy

The integration of new types of data sets created using new technologies should allow improvements and expansions to the curation and annotation approach established. For example, recent advances in long-read sequencing technologies^73,74^ should resolve genomic regions that could not be assembled previously using short-read technologies, thus significantly reducing the need for sequence curation. More contiguous assemblies would allow for improved identification, classification and functional annotation of inferred protein sequences.

Other improvements in functional annotation could also be achieved by employing a systems biology approach, integrating transcriptomic, proteomic and metabolomic data into the annotation process. For instance, information on pathways, essentiality and excretory/secretory (ES) proteins inferred from proteomic and metabolomic data sets could be integrated into the workflow, to add further experimental evidence to the computational predictions. However, the new, diverse and large data sets produced would need to be integrated into the existing data. Thus, experimental investigations should be followed by technical improvements and extensions. The establishment of a flexible, user-friendly and expandable platform that would facilitate the integration of such data sets would be desirable. Clearly, the present strategy provides a solid framework for further technical developments in this direction.

Other possible expansions include improvements of the speed, accuracy and/or efficiency of currently applied methods. For example, the functionality of the kinase-specific InterPro Domain Architecture (IDA) search tool, which has been established and employed in recent studies^27,28^ (Fig. 2), could be expanded to include all domain architectures represented in the InterPro database.

Furthermore, for all steps that employ HMM searches against sequence databases (including those carried out in InterProScan and specialised classification tools), there is also potential to increase search speed, sensitivity and accuracy by replacing the current search strategy with “HMM *versus* HMM” alignments (as implemented in the program HHblits^75^). However, such an expansion would require an adaptation of all third-party tools and the construction of customised databases of query sequence data sets (e.g., parasite proteomes) for use with HHblits. Given that “sequence *versus* HMM” searches perform very well for protein kinase catalytic domains, an upgrade to a “HMM *versus* HMM” alignment-based method might be considered when applying the present strategy to other protein families in the future.

In contrast, the structural prediction using I-TASSER could be readily automated and scaled to allow high-throughput analyses. The challenge here would be the requirement for substantial computing infrastructure and time. For example, computations of inferred kinase structures were run on an IBM iDataplex x86 system containing 67 nodes with 256 GB RAM and 16 cores per node (https://www.melbournebioinformatics.org.au/capabilities/), which led to a restriction of this approach to a subset of kinases^27,28^.

Taken together, our current strategy employs third-party tools including InterProScan, OrthoMCL, BLAT, CAP3, Exonerate, hmmalign^76^, MrBayes, Kinannote and I-TASSER (Fig. 2) and scripts developed by us (written in the programming languages Perl, bash and R; available from https://github.com/vetscience/kinase-gene-curation) that must be run in succession by the user, which requires relatively advanced bioinformatic skills. The major advantage of a workflow that is separated into multiple well-defined steps, is that it provides flexibility that could not easily be achieved using a more restrictive (i.e. allowing for less interaction and decision-making by the user), stand-alone version. Given that genomic and transcriptomic data sets of parasitic organisms are often highly diverse and that each project requires a careful assessment of data and subsequent selection of analysis strategies, an interactive, stepwise workflow represents a considerable advantage. To retain this flexibility and simultaneously create a more user-friendly application, a framework that allows the modularisation of individual steps into compatible and interchangeable components could be developed in the future. For instance, the module for kinase annotation could then simply be replaced with a module designed for a different protein group or family.

This extension might readily be achieved for well-defined enzyme classes such as phosphatases^77^; other less well-understood and studied classes might be more challenging. For instance, proteins of the cysteine-rich secretory proteins/antigen 5/pathogenesis-related 1 (CAP) superfamily (also called SCP/TAPS proteins)^78,79^ form an assembly of very diverse proteins with different domain architectures, which bears challenges regarding the functional annotation and classification of members of this superfamily^79^. For such a complex group of proteins, a library of subfamily-specific HMMs could be built based on functional domain architectures, as employed previously for atypical protein kinase families and subfamilies^27,28^. A module representing such a library could then be integrated into the established strategy. Taken together, we believe that the approach that we have established should be broadly applicable to a wide range of protein families.

## Conclusions

The bioinformatic strategy described and discussed here achieves comprehensive curation of kinase gene families and reliable annotation of the proteins that they encode, providing a powerful platform to explore the fundamental kinase biology of eukaryotic organisms and evolutionary relationships. Establishing a modular, robust and reproducible workflow that is generally applicable to the analysis of a wide range of protein families, from any organism, will likely be a useful tool to support systems biological investigations.

### Data availability

All computer code and data associated with this work is available from https://github.com/vetscience/kinase-gene-curation.

## References

[1] Mardis ER (2008). Next-generation DNA sequencing methods. Annu. Rev. Genomics Hum. Genet..

[2] Schuster SC (2008). Next-generation sequencing transforms today’s biology. Nat. Methods.

[3] Metzker ML (2010). Sequencing technologies - the next generation. Nat. Rev. Genet..

[4] van Dijk EL, Auger H, Jaszczyszyn Y, Thermes C (2014). Ten years of next-generation sequencing technology. Trends Genet..

[5] Goodwin S, McPherson JD, McCombie WR (2016). Coming of age: ten years of next-generation sequencing technologies. Nat. Rev. Genet..

[6] Korhonen PK, Young ND, Gasser RB (2016). Making sense of genomes of parasitic worms: Tackling bioinformatic challenges. Biotechnol. Adv..

[7] Mudge JM, Harrow J (2016). The state of play in higher eukaryote gene annotation. Nat. Rev. Genet..

[8] Durbin, R., Eddy, S. R., Krogh, A. & Mitchison, G. Biological sequence analysis: probabilistic models of proteins and nucleic acids. (Cambridge University Press, 1998).

[9] Finn RD (2010). The Pfam protein families database. Nucleic Acids Res..

[10] Xu Q, Dunbrack RL (2012). Assignment of protein sequences to existing domain and family classification systems: Pfam and the PDB. Bioinformatics.

[11] Altschul SF (1997). Gapped BLAST and PSI-BLAST: a new generation of protein database search programs. Nucleic Acids Res..

[12] Camacho C (2009). BLAST+: architecture and applications. BMC Bioinformatics.

[13] Koonin, E. V. & Galperin, M. Y. Genome annotation and analysis in *Sequence - evolution - function: computational approaches in comparative genomics*. 193–226 (Kluwer Academic Publishers, 2003). 21089240

[14] Schnoes AM, Brown SD, Dodevski I, Babbitt PC (2009). Annotation error in public databases: misannotation of molecular function in enzyme superfamilies. PLoS Comput. Biol..

[15] Eddy SR (1996). Hidden Markov models. Curr. Opin. Struct. Biol..

[16] Krogh A, Brown M, Mian IS, Sjölander K, Haussler D (1994). Hidden Markov models in computational biology. Applications to protein modeling. J. Mol. Biol..

[17] Henikoff JG, Henikoff S (1996). Using substitution probabilities to improve position-specific scoring matrices. Comput. Appl. Biosci..

[18] Martin DM, Miranda-Saavedra D, Barton GJ (2009). Kinomer v. 1.0: a database of systematically classified eukaryotic protein kinases. Nucleic Acids Res..

[19] Goldberg JM (2013). Kinannote, a computer program to identify and classify members of the eukaryotic protein kinase superfamily. Bioinformatics.

[20] Sonnhammer EL, Eddy SR, Durbin R (1997). Pfam: a comprehensive database of protein domain families based on seed alignments. Proteins.

[21] Alkan C, Sajjadian S, Eichler EE (2011). Limitations of next-generation genome sequence assembly. Nat. Methods.

[22] Nagy A (2008). Identification and correction of abnormal, incomplete and mispredicted proteins in public databases. BMC Bioinformatics.

[23] Nagy A (2011). Reassessing domain architecture evolution of metazoan proteins: major impact of gene prediction errors. Genes (Basel).

[24] Gilabert A, Curran DM, Harvey SC, Wasmuth JD (2016). Expanding the view on the evolution of the nematode dauer signalling pathways: refinement through gene gain and pathway co-option. BMC Genomics.

[25] Torres-Oliva M, Almudi I, McGregor AP, Posnien N (2016). A robust (re-)annotation approach to generate unbiased mapping references for RNA-seq-based analyses of differential expression across closely related species. BMC Genomics.

[26] Stroehlein AJ (2015). Defining the *Schistosoma haematobium* kinome enables the prediction of essential kinases as anti-schistosome drug targets. Sci. Rep..

[27] Stroehlein AJ (2017). Whipworm kinomes reflect a unique biology and adaptation to the host animal. Int. J. Parasitol..

[28] Stroehlein AJ (2016). Analyses of compact *Trichinella* kinomes reveal a MOS-like protein kinase with a unique N-terminal domain. G3 (Bethesda).

[29] Stroehlein AJ (2015). The *Haemonchus contortus* kinome - a resource for fundamental molecular investigations and drug discovery. Parasit. Vectors.

[30] Howe KL, Bolt BJ, Shafie M, Kersey P, Berriman M (2017). WormBase ParaSite - a comprehensive resource for helminth genomics. Mol. Biochem. Parasitol..

[31] Manning, G. Genomic overview of protein kinases. *WormBoo*k, ed. The *C. elega*ns Research Community, WormBook, https://doi.org/10.1895/wormbook.1.60.1 (2005). 10.1895/wormbook.1.60.1 PMC4780929 18050405

[32] Scheeff ED, Bourne PE (2005). Structural evolution of the protein kinase-like superfamily. PLoS Comput. Biol..

[33] Taylor SS, Kornev AP (2011). Protein kinases: evolution of dynamic regulatory proteins. Trends Biochem. Sci..

[34] Hunter T, Plowman GD (1997). The protein kinases of budding yeast: six score and more. Trends Biochem. Sci..

[35] Manning G, Whyte DB, Martinez R, Hunter T, Sudarsanam S (2002). The protein kinase complement of the human genome. Science.

[36] Cohen P (2002). Protein kinases - the major drug targets of the twenty-first century?. Nat. Rev. Drug Discov..

[37] Cohen P, Alessi DR (2013). Kinase drug discovery - what’s next in the field?. ACS Chem. Biol..

[38] Wu P, Nielsen TE, Clausen MH (2016). Small-molecule kinase inhibitors: an analysis of FDA-approved drugs. Drug Discov. Today.

[39] Dissous C, Ahier A, Khayath N (2007). Protein tyrosine kinases as new potential targets against human schistosomiasis. BioEssays.

[40] Dissous, C. *et al*. Receptor tyrosine kinase signaling and drug targeting in schistosomes in *Protein phosphorylation in parasites* (eds Doerig, C., Spaeth, G. & Wiese, M.) 337–356 (Wiley-Blackwell, 2013).

[41] Taylor CM (2013). Using existing drugs as leads for broad spectrum anthelmintics targeting protein kinases. PLoS Pathog..

[42] Hanks SK, Hunter T (1995). Protein kinases 6. The eukaryotic protein kinase superfamily: kinase (catalytic) domain structure and classification. FASEB J..

[43] Harris TW (2014). WormBase 2014: new views of curated biology. Nucleic Acids Res..

[44] Mi H, Muruganujan A, Casagrande JT, Thomas PD (2013). Large-scale gene function analysis with the PANTHER classification system. Nat. Protoc..

[45] Gough J, Karplus K, Hughey R, Chothia C (2001). Assignment of homology to genome sequences using a library of hidden Markov models that represent all proteins of known structure. J. Mol. Biol..

[46] Jones P (2014). InterProScan 5: genome-scale protein function classification. Bioinformatics.

[47] Kent WJ (2002). BLAT - the BLAST-like alignment tool. Genome Res..

[48] Huang X, Madan A (1999). CAP3: A DNA sequence assembly program. Genome Res..

[49] Li L, Stoeckert CJ, Roos DS (2003). OrthoMCL: identification of ortholog groups for eukaryotic genomes. Genome Res..

[50] Rice P, Longden I, Bleasby A (2000). EMBOSS: the European Molecular Biology Open Software Suite. Trends Genet..

[51] Slater GS, Birney E (2005). Automated generation of heuristics for biological sequence comparison. BMC Bioinformatics.

[52] Martin-Duran JM, Ryan JF, Vellutini BC, Pang K, Hejnol A (2017). Increased taxon sampling reveals thousands of hidden orthologs in flatworms. Genome Res..

[53] Campos TD (2014). Identification of G protein-coupled receptors in *Schistosoma haematobium* and *S. mansoni* by comparative genomics. Parasit. Vectors.

[54] Nor B (2016). Pipeline for the identification and classification of ion channels in parasitic flatworms. Parasit. Vectors.

[55] Roy A, Kucukural A, Zhang Y (2010). I-TASSER: a unified platform for automated protein structure and function prediction. Nat. Protoc..

[56] Kanehisa M, Goto S (2000). KEGG: Kyoto encyclopedia of genes and genomes. Nucleic Acids Res..

[57] Blum T, Briesemeister S, Kohlbacher O (2009). MultiLoc2: integrating phylogeny and Gene Ontology terms improves subcellular protein localization prediction. BMC Bioinformatics.

[58] Ronquist F (2012). MrBayes 3.2: efficient Bayesian phylogenetic inference and model choice across a large model space. Syst. Biol..

[59] Dunne MP, Kelly S (2017). OrthoFiller: utilising data from multiple species to improve the completeness of genome annotations. BMC Genomics.

[60] Dragan MA, Moghul I, Priyam A, Bustos C, Wurm Y (2016). GeneValidator: identify problems with protein-coding gene predictions. Bioinformatics.

[61] Nagy A, Patthy L (2013). MisPred: a resource for identification of erroneous protein sequences in public databases. Database (Oxford).

[62] Nagy A, Patthy L (2014). FixPred: a resource for correction of erroneous protein sequences. Database (Oxford).

[63] MacManes, M. D. The Oyster River Protocol: a multi assembler and kmer approach for *de novo* transcriptome assembly. *bioRxiv* http://dx.doi.org/10.1101/177253 (2017). 10.7717/peerj.5428 PMC6078068 30083482

[64] Venturini, L., Caim, S., Kaithakottil, G. G., Mapleson, D. L. & Swarbeck, D. Leveraging multiple transcriptome assembly methods for improved gene structure annotation. *bioRxiv* http://dx.doi.org/10.1101/216994 (2017). 10.1093/gigascience/giy093 PMC6105091 30052957

[65] Thorvaldsdóttir H, Robinson JT, Mesirov JP (2013). Integrative Genomics Viewer (IGV): high-performance genomics data visualization and exploration. Brief. Bioinform..

[66] Holt C, Yandell M (2011). MAKER2: an annotation pipeline and genome-database management tool for second-generation genome projects. BMC Bioinformatics.

[67] Bernot, A. Genome, transcriptome and proteome analysis. (John Wiley & Sons, Ltd., 2004)

[68] Coghlan, A. Nematode genome evolution. *WormBoo*k, ed. The *C. elega*ns Research Community, WormBook, https://doi.org/10.1895/wormbook.1.15.1 (2005). 10.1895/wormbook.1.15.1 PMC4781476 18050393

[69] Rödelsperger, C., Streit, A. & Sommer, R. J. Structure, function and evolution of the nematode genome. *eLS* https://doi.org/10.1002/9780470015902.a0024603 (2013).

[70] Bateman A (2010). Curators of the world unite: the International Society of Biocuration. Bioinformatics.

[71] Putman, T. E. *et al*. WikiGenomes: an open web application for community consumption and curation of gene annotation data in Wikidata. *Database (Oxford)*10.1093/database/bax025 (2017). 10.1093/database/bax025 PMC5467579 28365742

[72] Lee E (2013). Web Apollo: a web-based genomic annotation editing platform. Genome Biol..

[73] Roberts RJ, Carneiro MO, Schatz MC (2013). The advantages of SMRT sequencing. Genome Biol..

[74] Reuter JA, Spacek DV, Snyder MP (2015). High-throughput sequencing technologies. Mol. Cell.

[75] Remmert M, Biegert A, Hauser A, Soding J (2011). HHblits: lightning-fast iterative protein sequence searching by HMM-HMM alignment. Nat. Methods.

[76] Eddy SR (2011). Accelerated profile HMM searches. PLoS Comput. Biol..

[77] Chen MJ, Dixon JE, Manning G (2017). Genomics and evolution of protein phosphatases. Sci. Signal.

[78] Cantacessi C (2009). A portrait of the “SCP/TAPS” proteins of eukaryotes - developing a framework for fundamental research and biotechnological outcomes. Biotechnol.

[79] Cantacessi C, Gasser RB (2012). SCP/TAPS proteins in helminths - where to from now?. Mol. Cell Probes.

